# Malaria patients in Nigeria: Data exploration approach

**DOI:** 10.1016/j.dib.2019.104997

**Published:** 2019-12-16

**Authors:** Nureni Olawale Adeboye, Olawale Victor Abimbola, Sakinat Oluwabukola Folorunso

**Affiliations:** a Department of Mathematics & Statistics, Federal Polytechnic Ilaro, P.M.B. 50 Ilaro, Ogun State, Nigeria; b Department of Mathematical Sciences, Olabisi Onabanjo University, Ago-Iwoye, Ogun State, Nigeria

**Keywords:** Headache, Logistic regression, Malaria, Mosquitoes

## Abstract

Malaria is a life threatening disease which is usually transmitted to people through the bite of infected female anopheles mosquitoes. However, this article deals with the data exploration of malaria symptoms reported by 337 patients attended to at Federal Polytechnic Ilaro Medical centre, Ogun State Nigeria. The study covers a period of four (4) weeks monitoring of patients attendance, their consultation with physician and malaria test results as compared to their claims of malaria infection. Logistic regression was used for the basic analysis of the dataset and it was discovered that people in the age range 38–47 years are mostly affected with malaria and that females are the most infected gender species with headache being the most significant symptom based on its Wald statistic value. This study strongly recommends the introduction of a long lasting malaria prevention scheme that cut across all categories of ages and genders within the Nigerian community, and that self-medication should be seriously warned against as most claims of malaria were not actually found to be true upon verification.

**Table:** Specifications Table

Subject	Medicine
Specific subject area	Epidemiological, Public health, Biostatistics
Type of data	Table, Text
How data were acquired	Unprocessed Secondary data collected from Federal polytechnic Ilaro Medical Centre
Data format	Raw and partially analysed
Experimental factors	Observation of different Malaria Symptoms and the result of each patients after been tested for malaria
Experimental features	Computational Analysis: Histogram, Bar-chart, Logistic regression analysis
Data source location	Federal Polytechnic Ilaro Medical Centre, Ilaro, Ogun State, Nigeria
Data accessibility	All the data are available in this data article as supplementary materials

**Table:** 

**Value of the Data** • The data on malaria infection could be useful for government and health workers to make decisions that would reduce the risk of malaria infection among the populace. • This work provides a deeper understanding of the prevalence and prognosis of malaria infection. • The data can be useful in malaria infection awareness, management and treatment. • The data could be used as a baseline for comparison in future studies. • The data reveals high significant impacts of prevalent factors such as headache, pain, fever, cold etc. on malaria morbidity

## 1 Data

The data set used in this article was collected as a secondary data from Federal Polytechnic Ilaro Medical centre, Ilaro Ogun state, Nigeria and it contains information on 337 patients who presented themselves for consultation on malaria related infections. The symptoms reported by the patients were recorded and information about the same patients were collected after been tested for malaria. These patients are between the ages of 3 and 77 years of whom 180 are females and 157 are males, and their data was collected for a period of 4 weeks. The recorded symptoms as reported by the patients were all compared with the results of the malaria test, and the results of the malaria test was used for the target variables.

This dataset consist of 15 malaria symptoms which are “Fever, Cold, Rigor, Fatigue, Headache, Bitter-tongue, Vomiting, Diarrhea, Convulsion, Anemia, Jaundice, Cocacola-Urine, Hypoglycemia, Prostration, and Hyperpyrexia” as collected. From the dataset, Ages of the patients are recorded in years while gender were encoded in ordinal form as “0” for Male and “1” for Female. Other features are encoded in integers (“0” for non-presence and “1” for the symptoms presence). This raw dataset which has been approved by the medical director, representing the institutional bioethics committee is available and can be assessed as Supplementary data.

Descriptive analyses were performed and logistic regression analysis was also used to describe and analyze the data set. The data is summarized under different classifications which are: classification based on gender (sex), malaria infection classification for age, classification of malaria infection by sex and classification based on some common malaria symptoms.

### 1.1 Analysis of age of the patients

The frequency table showing the analysis of the age of all the 337 patients is shown in Table 1. In Table 1, it can be seen that the mean age of the patients is 30.35 years, the minimum and maximum ages are 3 year and 77 years respectively. The data set is slightly positively skewed and leptokurtic with a coefficient of Skewness and kurtosis of 0.755 and 0.536 respectively.

**Table 1:** Analysis of age in years.

Statistics	
N	
Valid	337
Missing	0
Mean	30.35
Median	29.00
Mode	31
Std. Deviation	14.721
Variance	216.704
Skewness	.755
Std. Error of Skewness	.133
Kurtosis	.536
Std. Error of Kurtosis	.265
Range	74
Minimum	3
Maximum	77
Sum	10,227

**Fig. 1:**
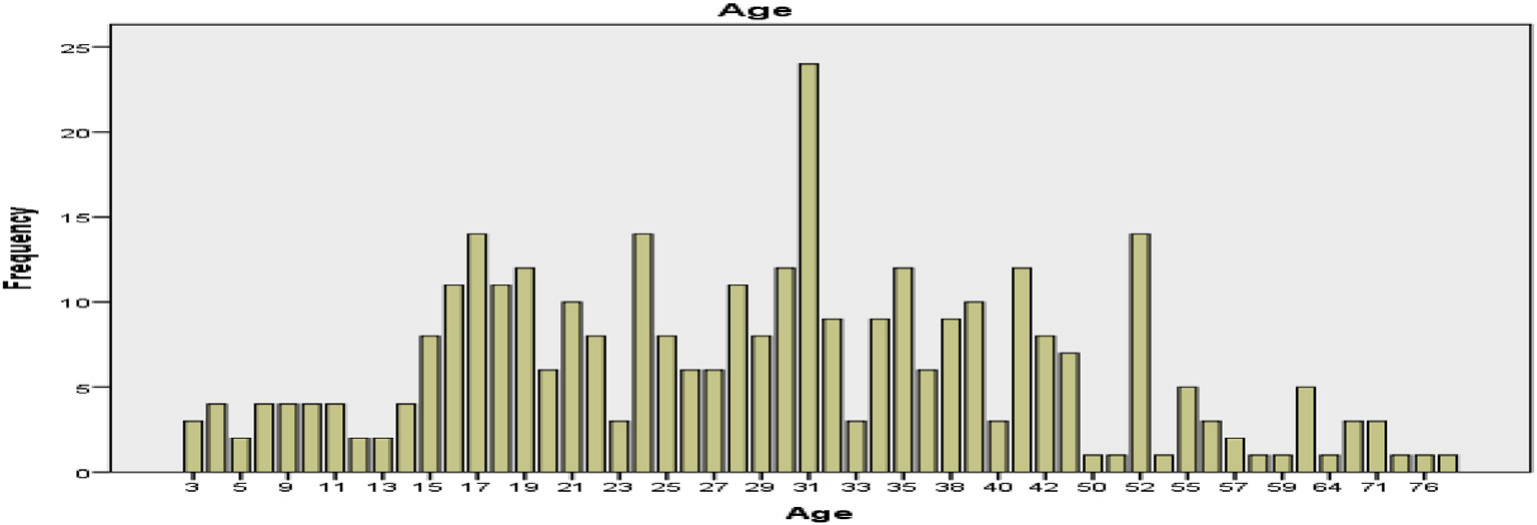
Age distribution (Years).

**Fig. 2:**
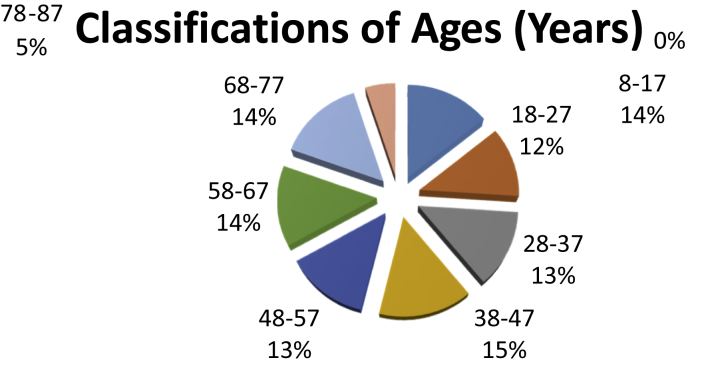
Percentage distribution of Ages (Years).

A diagrammatic representation of the age distribution and age range of the patients is as shown in Fig. 1, Fig. 2 respectively. The age of the patients were classified into eight different groups (or classes) and the respective frequencies are as shown in Table 2. It can be seen from Table 2 that majority (50) of the patients are in the age group 38–47 years which is approximately 15% of the total population. The diagrammatic representation of the information in Table 2 is as shown in Fig. 2.

**Table 2:** Classification of age of patients (Years).

Age Range	Frequencies	Percentage
8–17	48	14.2
18–27	40	11.8
28–37	44	13.0
38–47	50	14.8
48–57	44	13.0
58–67	46	13.6
68–77	49	14.5
78–87	17	5.0
Total	338	100

**Fig. 3:**
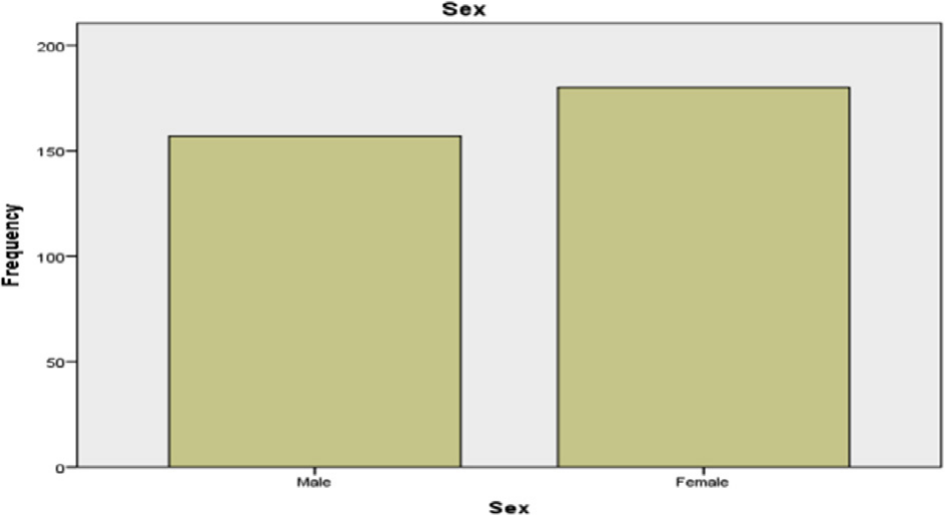
Bar Chart showing the distribution of gender.

Information on the gender is as shown in Table 3 and the respective frequencies are also displayed. From Table 3, it can be seen that most of the patients were female. The diagrammatic representation is as shown in Fig. 3.

**Table 3:** Distribution of gender of the patients.

Sex	Frequency	Percent	Valid Percent	Cumulative Percent
Male	157	46.6	46.6	46.6
Female	180	53.4	53.4	100.0
Total	337	100.0	100.0

### 1.2 Analysis on malaria diagnosis using logistic regression

**Fig. 4:**
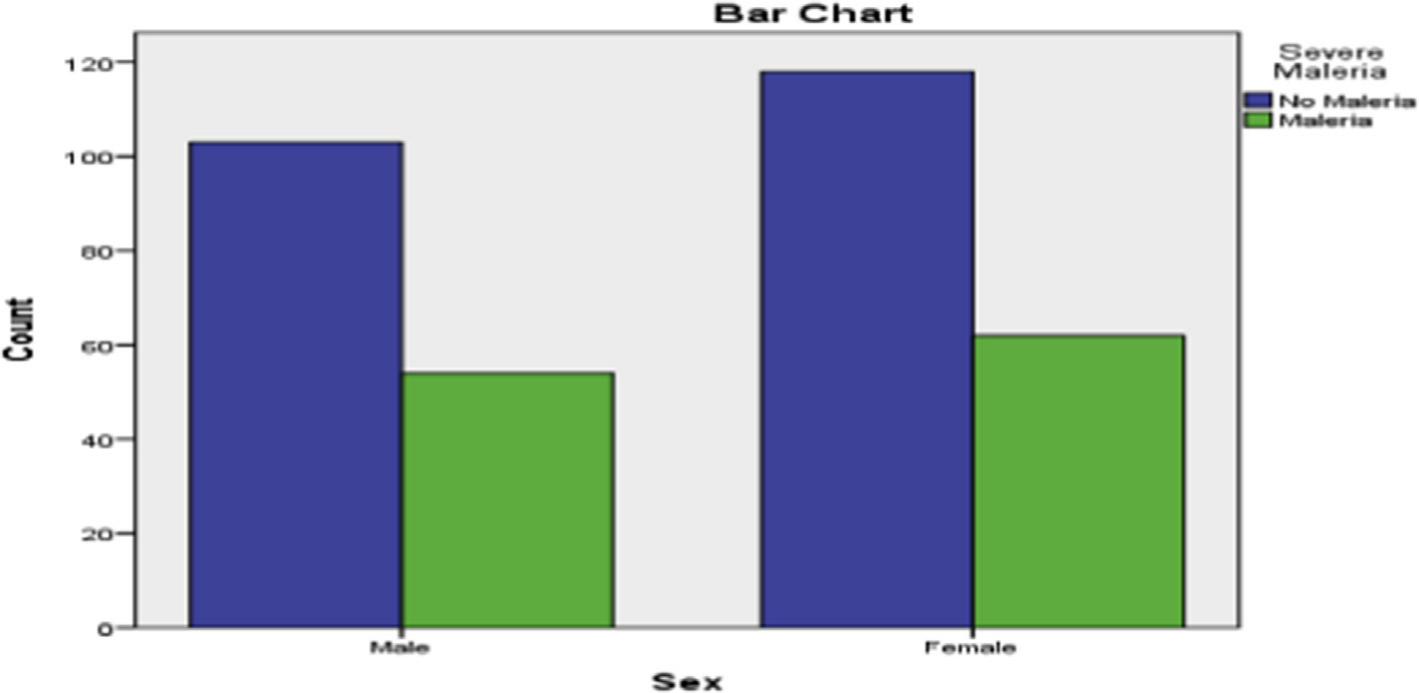
Multiple Bar Chart showing the distribution of gender and Malaria.

**Fig. 5:**
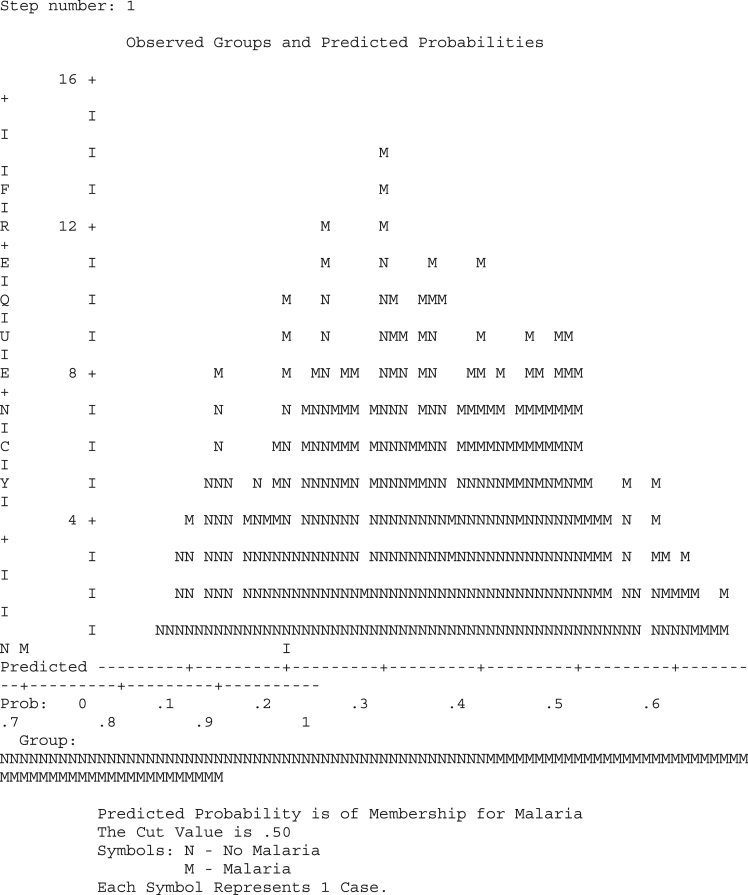
Diagram of predictive probabilities.

Information on the diagnosis of patients who presented themselves for malaria treatment was shown in Table 4 and it was observed that only 116 of the 337 reported cases were actually found to be infected with malaria, of which most of them are female. The diagrammatic representation of Table 4 is as shown in Fig. 4. It was observed that in Fig. 5, the chart of the predicted probabilities gave a Cut Value/threshold of 0.5 and the goodness of fit test was carried out using Hosmer and Lemeshow Test.

**Table 4:** Cross tabulation for gender and Malaria of patients.

Sex * Severe Malaria Cross tabulation			
Count			
	Severe Malaria		Total
	No Malaria	Malaria	
Sex			
Male	103	54	157
Female	118	62	180
Total	221	116	337

## 2 Experimental design, materials and methods

This article shows the strength of the significant level of the perceived as well as diagnosed malaria symptoms using logistic regression analysis. It equally examined the linear relationship between the malaria predicted binary classes. Research on malaria has been a great concerns to government and world health organizations. According to Ref. [1], there were estimated deaths of 435,000 from malaria globally in 2017, compared with 451,000 estimated deaths in 2016, and 607 000 in 2010.

According to researches, several aspect of malaria prediction method has been studied. And different forms of dataset have been used such as malaria cell image dataset and different forms of numerical dataset.

Artificial neural networks, Machine learning/Data mining and deep learning methods has been helpful to previous researchers in predicting malaria outbreak/infections in different regions and community all over the world. Some have gone as far as using geospatial based and weather based dataset in predicting malaria which has been a very huge success in previous years and different recommendation have been made [[1], [2], [3], [4], [5], [6], [7], [8], [9]].

Malaria is transmitted exclusively through the bites of Anopheles mosquitoes. The intensity of transmission depends on factors related to the parasite, the vector, the human host, and the environment. Symptoms of malaria include fever, headache, and vomiting, and other listed symptoms in the dataset which usually appear between 10 and 15 days after the mosquito bite. If not treated, malaria, more so falciparum malaria, can quickly become life-threatening by disrupting the blood supply to vital organs [[10], [11], [12], [13], [14]].

Chi-square test of independence can equally be used to analyze the data collected. For instance, a cross-tabulation of gender and Malaria outcome of the patients after been tested can be classified into contingency table as shown in Table 4. In this research however, logistic regression analysis was used to analyze the data set.

Table 5 shows the classification table at step 1.

**Table 5:** Classification Table.

	Observed	Predicted		
		Severe Malaria		Percentage Correct
		No Malaria	Malaria	
Step 1	Severe Malaria			
	No Malaria	204	17	92.3
	Malaria	91	25	21.6
	Overall Percentage			68.0

Table 6 shows the variables in the equation at Step 1.

**Table 6:** Variables in the equation.

		B	S.E.	Wald	Df	Sig.	Exp(B)
Step 0	Constant	−0.645	0.115	31.606	1	0.000	0.525

Table 7 shows the omnibus tests of model coefficients.

**Table 7:** Test of model coefficients.

Omnibus Tests of Model Coefficients				
		Chi-square	df	Sig.
Step 1	Step	29.301	17	.032
	Block	29.301	17	.032
	Model	29.301	17	.032

Table 8 shows the model summary using the log-likelihood, Cox & Snell R square and Negelkerke R square.

**Table 8:** Model summary.

Step	−2 Log likelihood	Cox & Snell R Square	Nagelkerke R Square
1	404.614^a^	0.083	0.115

a Estimation terminated at iteration number 4 because parameter estimates changed by less than .001.

Table 9 shows the Hosmer and Lemeshow Test.

**Table 9:** Hosmer and Lemeshow test.

Step	Chi-square	Df	Sig.
1	5.266	8	.729

Table 10 shows Contingency Table for Hosmer and Lemeshow Test.

**Table 10:** Contingency Table for Hosmer and Lemeshow test.

		Severe Maleria = No Malaria		Severe Maleria = Malaria		Total
		Observed	Expected	Observed	Expected	
Step 1	1	31	29.928	3	4.072	34
	2	25	27.468	9	6.532	34
	3	25	25.874	9	8.126	34
	4	24	24.318	10	9.682	34
	5	23	23.077	11	10.923	34
	6	26	21.622	8	12.378	34
	7	21	20.148	13	13.852	34
	8	17	18.659	17	15.341	34
	9	15	17.210	19	16.790	34
	10	14	12.696	17	18.304	31

Table 11 shows the classification table for all the step 1.

**Table 11:** Variables in the equation.

		B	S.E.	Wald	df	Sig.	Exp(B)	95% C.I.for EXP(B)	
								Lower	Upper
Step 1^a^	Age	.013	.008	2.380	1	.123	1.013	.997	1.030
	sex (1)	−.076	.250	.092	1	.761	.927	.567	1.514
	fever (1)	.023	.287	.006	1	.937	1.023	.583	1.795
	cold (1)	−.345	.253	1.856	1	.173	.708	.431	1.163
	rigor (1)	−.182	.257	.502	1	.478	.833	.503	1.380
	fatigue (1)	−.267	.252	1.117	1	.290	.766	.467	1.256
	headace (1)	−.795	.286	7.703	1	.006	.452	.258	.792
	bitter_tongue (1)	.187	.250	.558	1	.455	1.205	.738	1.967
	vomitting (1)	−.034	.480	.005	1	.944	.967	.377	2.479
	diarrhea (1)	−.478	.254	3.535	1	.060	.620	.377	1.020
	Convulsion (1)	.423	.262	2.614	1	.106	1.527	.914	2.549
	Anemia (1)	.033	.257	.016	1	.898	1.033	.625	1.710
	jundice (1)	−.139	.261	.285	1	.593	.870	.522	1.450
	cocacola_urine (1)	−.377	.248	2.304	1	.129	.686	.422	1.116
	hypoglycemia (1)	−.772	.396	3.806	1	.051	.462	.213	1.004
	prostraction (1)	.603	.315	3.671	1	.055	1.828	.986	3.388
	hyperpyrexia (1)	−.017	.362	.002	1	.962	.983	.483	1.999
	Constant	−.619	.767	.650	1	.420	.539		

a Variable(s) entered on step 1: age, sex, fever, cold, rigor, fatigue, headace, bitter_tongue, vomitting, diarrhea, Convulsion, Anemia, jundice, cocacola_urine, hypoglycemia, prostraction, hyperpyrexia.

Fig. 5 shows the diagram of predictive probabilities.

## Figures and Tables

**Fig. S1:**
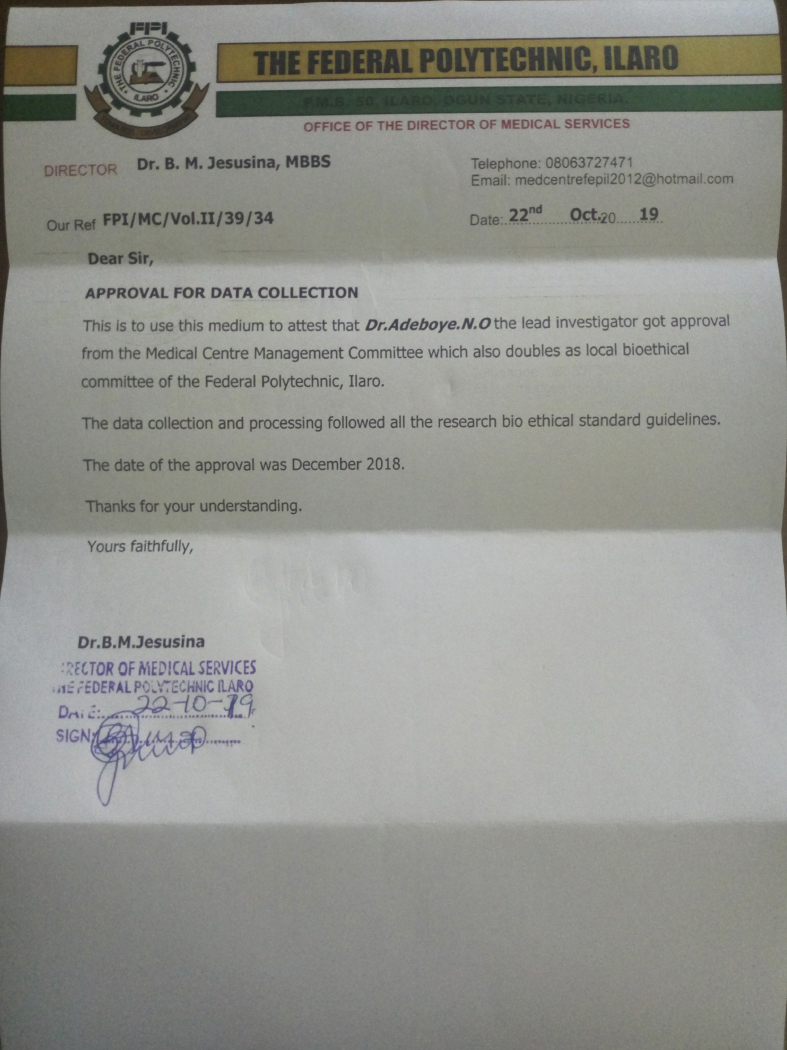


## References

[1] World Health Organization (2018). This Year's World Malaria Report at a Glance. https://www.who.int/malaria/media/world-malaria-report-2018/en/.

[2] National Aeronautics and Space Administration (2017). Using NASA Satellite Data to Predict Malaria Outbreak. https://www.nasa.gov/feature/goddard/2017/using-nasa-satellite-data-to-predict-malaria-outbreaks.

[3] KobinaPaintsil E., Omari-Sasu A.Y., Addo M.G., AkwasiBoateng M. (2019). Analysis of Haematological parameters as predictors of malaria infection using a logistic regression model: a case study of a hospital in the ashanti region of Ghana. Malar. Res. Treat..

[4] Maina R.N., Walsh D., Gaddy C. (2010). Impact of plasmodium falciparum infection on hematological parameters in children living in Western Kenya. Malar. J..

[5] Nankabirwa J., Brooker S.J., Clarke S.E. (2014). Malaria in school-age children in Africa: an increasingly important challenge. Trop. Med. Int. Health.

[6] Ugwu C.L.J., Zewotir T.T. (2018).

[7] MuthiiMuriuki J., Kitala P., Muchemi G., Njeru L., Karanja J., Bett B. (2016). https://www.ncbi.nlm.nih.gov/pmc/articles/PMC4982001.

[8] Sharma V., Kumar A., Panat L., Karajkhede G. (2015). https://www.researchgate.net/publication/29108473/.

[9] Darkoh E.L., AseiduLarbi J., AdjeiLawer E. (2017). A Weather-Based Prediction Model of Malaria Prevalence in Amenfi West District, Ghana, Malaria Research and Treatment.

[10] Adeboye N.O., D Ezekiel I. (2018). On time domain analysis of malaria morbidity in Nigeria. Am. J. Appl. Math. Stat..

[11] Reeder J.C. (2001). Towards a malaria vaccine for Papua New Guinea. P. N. G. Med. J..

[12] Gerristsen A., Kruger P., Van der Leo M., Grobusch M. (2008). Malaria incidence in limpopo province, South Africa, 1998-2007. Malar. J..

[13] Ayeni A.O. (2011). Malaria morbidity in Akure, Southwest Nigeria: a temporal observation in climate change scenario. Trends Appl. Sci..

[14] Korenromp E., Kiniboro B. (2007). http://www.W.H.O.Int/malaria/publications/atoz/incidence_estimation.

